# The wicked problem of healthcare student attrition

**DOI:** 10.1111/nin.12294

**Published:** 2019-05-06

**Authors:** Claire Hamshire, Kirsten Jack, Rachel Forsyth, A. Mark Langan, W. Edwin Harris

**Affiliations:** ^1^ Faculty of Health, Psychology and Social Care Manchester Metropolitan University Manchester UK; ^2^ Centre for Excellence in Learning and Teaching Manchester Metropolitan University Manchester UK; ^3^ Faculty of Science and Engineering Manchester Metropolitan University Manchester UK

**Keywords:** midwifery, nurse education, retention, undergraduates, wicked problems, withdrawal

## Abstract

The early withdrawal of students from healthcare education programmes, particularly nursing, is an international concern and, despite considerable investment, retention rates have remained stagnant. Here, a regional study of healthcare student retention is used as an example to frame the challenge of student attrition using a concept from policy development, wicked problem theory. This approach allows the consideration of student attrition as a complex problem derived from the interactions of many interrelated factors, avoiding the pitfalls of small‐scale interventions and over‐simplistic assumptions of cause and effect. A conceptual framework is proposed to provide an approach to developing actions to reduce recurrent investment in interventions that have previously proved ineffective at large scale. We discuss how improvements could be achieved through integrated stakeholder involvement and acceptance of the wicked nature of attrition as a complex and ongoing problem.

## 1 INTRODUCTION

Student attrition is a costly challenge for higher education (Beer & Lawson, 2017), and voluntary early withdrawal of midwifery and nursing students is a concerning trend (Dante, Petrucci, & Lancia, 2013; Hughes, 2013). Research across the higher education sector has identified that a broad range of factors can impact student success, with the most frequently cited barriers being personal issues, financial problems and academic difficulties (Cameron, Roxburgh, Taylor, & Lauder, 2011a, 2011b; Lancia, Petrucci, Giorgi, Dante, & Cifone, 2013; Tinto, 1994; Urwin et al., 2010; Yorke, 2004). While single factors can be mitigated at a local level (e.g., that of the institution or programme), the range of factors impacting student decisions makes it difficult to predict student success and retention. Thus, identifying and targeting appropriate interventions to support student progression remains a challenge for which there is no easy solution (Jones‐Schenk & Harper, 2014).

The high rate of healthcare student attrition has generated a significant body of international research over several decades (see, for instance, the reviews by Merkley, 2015; Mulholland, Anionwu, Atkins, Tappern, & Franks, 2008), underscoring the widespread nature of the problem. While a variety of factors have been identified as contributing to healthcare student attrition, the clearest finding is that the factors are numerous and that they interact with one another in complex ways (Sabin, 2012). Constraints such as local resource allocation and political policy change have added to the challenge of mitigating drivers of healthcare student attrition. Hamshire, Barrett, Langan, Harris, and Wibberley (2017) demonstrated that despite considerable investment in actions designed to address particular issues, such as targeted personal support, campus‐based supporting structures and managing expectations and experiences of placements, there is little evidence that these efforts significantly impact overall student retention. There is consensus that addressing the problem requires flexible and inclusive approaches to overcome the lack of success of simple solutions (Harris, Vanderboom, & Hughes, 2009). Educators therefore need to confront the drivers of attrition (Abele, Penprase, & Ternes, 2013) and apply processes that involve the consideration of multiple interacting systems to help to interrogate the evidence and propose solutions. Approaches for addressing student attrition need a shift in thinking, to recognise this complexity and to manage the potential consequence of interventions.

One approach to managing large, complex problems is to consider solutions in the context of the ‘wicked problem’ framework (Rittel & Webber, 1973), which was specifically proposed to address problems that are difficult to manage. Wicked problems are characterised as dynamic, complex and impossible to solve, with simple solutions addressing only one dimension of the whole (Sherman & Peterson, 2009). These properties distinguish wicked problems from ‘tame’ problems’, for which a clear problem and workable solutions can be identified (Varpio, Aschenbrener, & Bates, 2017). Wicked approaches conceptualising complex issues and developing policies have been proposed for many areas of enquiry, for example in public policy‐making (McGrandle & Ohemeng, 2017; Sherman & Peterson, 2009) and in environmental conservation (DeFries & Nagendra, 2017). In this paper, the wicked problem approach is applied to the challenge of student attrition in healthcare education, using data from a cross‐sectional study.

## 2 THE WICKED PROBLEM FRAMEWORK

For wicked problems, the use of the word ‘wicked’ is an indication of the complexity, importance and persistence of a problem, rather than an indication of aberrance (Coyne, 2005). Wicked problem solutions have competing and changing requirements, and involve many stakeholders, each with their own values and priorities (Harris et al., 2009). Stakeholder views will typically be shaped by both personal and professional characteristics, influencing how they explore and address causal factors contributing to wicked problems and the validity of solutions (Roberts, 2000). There has been interest in using the framework of wicked problems in diverse policy areas such as educational quality (Jordan, Kleinsasser, & Roe, 2014; Krause, 2012), mental health (Hannigan & Coffey, 2011; Harris et al., 2009) and curriculum design (Hawick, Cleland, & Kitto, 2017). Such approaches conceptualise wicked problem solutions as a process, focussing on continuous problem‐solving and evaluation, rather than focussing on short‐term outcomes.

The framework for mitigating a wicked problem was laid out by Rittel and Webber (1973), with consideration to problems, solutions and stakeholders. The structure of a wicked problem is, by definition, difficult to identify and may change through time or be different in different contexts. As a consequence, it follows that solutions may not have clear outcomes or stopping points. Because of these properties, it is argued that the context of wicked problems should constitute aspects of process and of policy, so that formulation of the problem is revisited along with efficacy and changing effectiveness of actions to alleviate the problem. Perhaps the most challenging aspect of wicked problems, however, is the fact that different stakeholder segments may agree, disagree or even fail to perceive important aspects of both the problem and of solutions, and can vary in the complexity of stakeholder relationships (Alford & Head, 2017).

## 3 THE PROBLEM OF HEALTHCARE STUDENT ATTRITION

The complex blend of stakeholders associated with healthcare education results from students working both within universities and in publicly funded clinical environments. In the United Kingdom (UK), the competencies required to register as a qualified Nurse are stated by the Nursing & Midwifery Council (NMC, 2010). Education is split equally between the University and clinical environment, which is similar to the approach across Europe and other countries such as the United States and Australia (Saarikoski, Marrow, Abreu, Riklikiene, & Özbicakçi, 2007). The high rate of withdrawal from UK Nursing courses is a matter of national interest; about a quarter of those enrolled on Nursing courses do not go on to qualify as nurses (Mulholland et al., 2008).

Hamshire, Willgoss, and Wibberley (2013) explored reasons why UK students considered leaving pre‐registration courses in nursing and allied healthcare programmes. Around a thousand online survey responses from students across healthcare courses and year cohorts showed that almost half had considered leaving. Three distinct themes emerged to explain this: dissatisfaction with high academic workload and poor academic support; difficulties associated with clinical placements; and personal concerns and challenges. A large number of students identified a combination of reasons for leaving (within and across themes). Key factors that influenced the decision to continue their studies included support from family and friends, personal determination, interesting and enjoyable placements, and support from staff. The outcomes of this example study led to considerable investment in improving students’ experiences, including implementation of peer mentoring schemes and a variety of specialised first‐year support courses. However, there appeared to be little change in students’ perceptions when the survey was repeated 4 years later with new cohorts on the same courses (Hamshire et al., 2017; Jack et al., 2018). This lack of improvement in students’ qualification rates has been noted by others, for example Varpio et al. (2017) who suggested that the challenges for educators in some health professions are so complex that they defy resolution. With the increasing complexity in healthcare systems, changes in policy and practice in one area will inevitably affect the workplace elsewhere, sometimes with unexpected results that appear impossible to undo (Hannigan & Coffey, 2011; Harris et al., 2009).

## 4 THE WICKED PROBLEM OF STUDENT ATTRITION

In terms of terminology, there is a variety of measurements associated with the completion academic studies, such as ‘retention’, ‘withdrawal’, ‘timely completion’, ‘discontinuation’, ‘non‐completion’, ‘survival’, ‘graduate completion’ and ‘student success rate’. In his seminal work on attrition, Tinto focused on first‐year withdrawal (Tinto, 1994; Tinto & Goodsell, 1993). Currie et al. (2014) provided numerous descriptions including failing to enrol, enrolling but failing to attend class and so on. In the UK, student retention generally refers to the extent to which learners remain within a higher education institution, completing a programme of study in a pre‐determined time period (Jones, 2008). In terms of interpretation of student withdrawal, it is noteworthy that in the overwhelming majority of cases, student attrition has been framed as an institutional failing, and there is a motive to document studies into retention in a manner that contextualises it in the least detrimental way as possible to the institution. However, it should be acknowledged attrition is not always a negative outcome and withdrawal can be a positive choice for individual students (Boyd & Mckendry, 2012). This outcome positions personal needs at odds with the intentions of the education providers, potentially complicating the messages about withdrawal from the student stakeholder position.

Student attrition in general should be considered to be a dynamic problem, with non‐linear responses to external influences that vary across place and time. There are several large‐scale, multi‐institution, longitudinal studies that have documented factors associated with student withdrawal (e.g., Yorke & Longden, 2008), and a number of integrative literature reviews (e.g., Pitt, Powis, Levett‐Jones, and Hunter (2012). However, the majority of research into interventions to alleviate student attrition is founded on small‐scale studies at single institutions (Cameron et al., 2011b). As a consequence, the proposed solutions are generally specific to a particular context and should be treated with a degree of caution, as they may not be transferrable across settings. Previous research highlights complex reasons that underpin withdrawal by identifying multiple, interacting factors associated with the probability of leaving. Tinto (1975) argued that student achievement is determined largely by integration into both the social and academic aspects of an institution. That is, the likelihood of whether students will continue is dependent on integration during learning transitions (Tinto & Goodsell, 1993).

## 5 THE PARTICULARLY WICKED PROBLEM OF HEALTHCARE STUDENT ATTRITION

Table 1 maps Rittel and Webber's (1973) properties of a wicked problem to the characteristics of healthcare student attrition. As described, healthcare student success in tertiary education has many interested stakeholders, which include the students, clinical professionals and policy‐makers. There are international dimensions to the courses and their content and also drivers of withdrawal that can emanate from home campus provision, clinical placement providers or personal circumstances. Examining attrition through the lens of a wicked problem supports in‐depth exploration of the multiple influences on attrition and how individual solutions to specific problems are not always effective. Further, strategies to reduce attrition in healthcare students need to consider the student lifecycle over time, from recruitment to graduation, in partnership with all stakeholders, to set reasonable student expectations ensuring that a career within healthcare is both desired and valued (Fontaine, 2014).

**Table 1:** Rittel and Webber's (1973) properties of a Wicked Problem (adapted from McGrandle & Ohemeng, 2017, p. 231) in the context of healthcare student attrition

Characteristics of wicked problems	Attrition characteristics
No clear definition of the problem	Multiple stakeholders have differing definitions of the problem. For the HEI, student attrition can be costly due to loss of funding and impacts on reputation. For students, there may be impacts on well‐being, but ability to freely leave an unsuitable programme is not a problem; for healthcare providers, attrition from healthcare programmes is a problem in terms of workforce planning and supply. Various descriptions of the problem are unhelpful such as withdrawal, discontinuation and non‐completion. A tolerable level of attrition is difficult to define
Never ending solutions and amendments	Solutions proposed address particular issues such as personal support, placement experiences and academic achievement. All can be beneficial although do not address the complexity of the interaction between such individual problems and how this relates to student retention
No right or wrong evaluation or solution	As definitions of the problem vary, so will the associated evaluations and solutions. It is difficult to achieve the ‘right’ evaluation if the cause is interrelated and complex
No immediate test of solution	Testing solutions to student attrition are difficult as potentially there are far‐reaching consequences, which affect the multiple stakeholders in different ways. Due to the complex nature of attrition, testing an individual solution is difficult and can take many academic cycles to show impact
No trial and error phase	Changes to policies can take months of preparation and require the multiple stakeholders to develop the solutions. Changes are often costly and once actions (e.g., policies) are in place, these remain for some time
No criteria to know if all solutions have been identified	The nature of the problem may not be apparent or agreed and may change in time. The wide consequences of attrition might not ever be fully understood
Each wicked problem is unique	The problem of healthcare student attrition is a product of many localised features, therefore does not lend itself to generalised solutions
Wicked problems can be the result of another problem	Student attrition is the result of multiple factors that are complex and are interrelated. Each problem requires a different solution and could have knock‐on effects, making individual solutions difficult
Framing of the problem affects and limits potential solutions	Tame solutions have been proposed in the literature for isolated aspects of student attrition. However, these do not account for the complexity of the problem
Pressure on policy‐makers	There is intense pressure on policy‐makers to find solutions to the problem. The implications of attrition are costly and have far‐reaching consequences on multiple levels

Throughout their studies, healthcare students constantly undergo a process of transition as they adapt their expectations of both the higher education and the clinical practice environments. Those students who feel socially and academically integrated in both environments are more likely persist in their studies (Scanlon, Rowling, & Weber, 2007). The challenge for healthcare educators is to try to identify which factors will affect students’ experiences of this transition and respond in a positive and supportive way. The ability of any given student to complete their course is governed by many interacting factors, including the educational experiences of the student prior to enrolling; the social and academic engagement between the student, their peers and the institution; job certainty, national events and the commitment of the student to the institution (Urwin et al., 2010). In accord with the findings of Bryson and Hardy (2012), it is clear that the specificity of each student's personal situation defines their learning experience (Beer & Lawson, 2017). Definitive results from specific interventions may take up to 5 years to emerge (Cameron et al., 2011b), and despite a significant body of literature in this field, there is limited robust evaluation of specific interventions that are designed to reduce attrition. The complex nature of the student environment, comprising of many dynamic extrinsic and intrinsic factors governing their potential success in both academic and professional components of their courses, underpins our assertion that the problem of improving student achievement is wicked in nature.

## 6 KEY DRIVERS OF HEALTHCARE STUDENT WITHDRAWAL—A CROSS‐SECTIONAL STUDY

To illustrate the nature of the wicked problem of healthcare student retention problem, we draw on key outcomes of a repeat regional cross‐sectional study that identified factors contributing to attrition in nursing and healthcare students at nine institutions in the North West of England. Briefly, an online survey was made available to all undergraduate students studying on healthcare education programmes (*n* = ca. 20,000) at nine participating institutions; full details are reported in Hamshire et al. (2013, 2017). Ethical approval was obtained from the Manchester Metropolitan University Research and Ethics Committee.

The survey was run in both 2011 and in 2015, and consisted of four sections: demographic data, campus‐based learning, placement‐based learning and personal circumstances. One section of the survey enquired if students had considered leaving their course, and then invited those that had to provide open comments about the reasons that led them to leave or to continue on their programme.

In 2011, 1,080 students completed the first survey (response rate 5%), and in 2015, there were 1,983 respondents (response rate 10%). Demographic data showed that the sample in both cases was largely representative of the healthcare student body. Of the respondents, 77% were studying Nursing programmes and the largest group of respondents were in their first year of study.

The second survey demonstrated that there had been little change in students’ perceptions of their learning experiences despite considerable investment in a range of interventions by both higher education institutions and regional funding bodies (Hamshire et al., 2017). Reasons for considering withdrawal from their courses in the later survey were thematically analysed following the approaches of Ritchie and Spencer (2002). Over 42% (735) of the students reported that they had considered leaving their programme and 712 of these added comments regarding their thoughts about early withdrawal. Thematic analysis of the student comments related to the ‘Have you considered leaving?’ question identified three key themes as shown in Table 2, with examples of student comments.

**Table 2:** Exemplar student comments for the three identified themes in relation to early withdrawal

Factor influencing attrition	Example data
Concerns due to personal circumstances	We have less money to live off than other students over the year when we have an extra 2 months to fund. Watching other people live the university lifestyle while we're supposed to feel like uni students is like rubbing it in our facesIt's not a normal sort of degree. In regards to the intensity and hours. I have not been able to participate in sport the way I would have liked. Holidays and paid work in the summer is very limited as we only have a month offI have felt overwhelmed several times on the course, there is very little consideration given for those who have children with little childcare support especially regarding placement hours. When I approached a member of staff about this I was told that was just the way it is, which was very discouraging and unhelpful
Workload pressure	I have considered leaving the course due to the overlap of work placements and assignments. Throughout this academic year, all assignments have had a deadline that is when we are on placement, therefore it has been very difficult to complete assignments to the best of my abilities and I have been extremely tired on placement due to having late nights to complete assignmentsStresses of having so much academic work to do whilst being on placement—sometimes feeling like I'm working a full time job for zero pay and getting so stressed out that my own mental health suffered. I was close to quitting when I became ill from all the stress but I'm determined not to waste the rest of my life in rubbish jobs
Clinical placement culture	When you're on a placement and the staff treat you as a healthcare assistant it can really get you down, as staff constantly see you as an extra pair of hands. I honestly feel that student nurses are used to bridge the gap in the staffing shortages on most wards. And this problem needs to be addressed as it affects the student learning experience. And if student nurses try to broach this subject with staff we are often thrown back with the saying that we are to ‘posh to wash’, which is a complete lieNursing is fascinating, however the politics that go on in placement, the lack of doors open to be able to progress from nursing into higher positions and the lack of pay and expectations of nurses makes me think every single day I am on placement about leaving the course. The only thing keeping me on is that I have one year left and I would like to try and have a go at progressing from nursing. But it really scares me that I am going to get stuck as a band 5 [entry level job] like everybody elseThe way that nurses get treated by hospitals and the community from day one (first day as a student nurse) puts me off being in the profession as it doesn't seem to improve over time or as we qualify

Understanding the complexity of causative factors that impact on an individual student's learning experiences and thus engagement can be challenging (Currie et al., 2014). With a more traditional approach to problem‐solving, many of the challenges could appear as peripheral or outside the sphere of influence of the academic manager charged with addressing student retention. While one body is responsible for student funding, another may be responsible for a provider's approach to placement students, and another responsible for timetabling student placements and assignment deadlines. Evaluation of a funded project tends only to consider intended consequences and may not identify wider problems which the project has generated. For example, a course team may decide to provide early clinical placement experience to motivate students, but without considering the additional workload pressures for clinical staff in supervision of students without underpinning knowledge. This can lead to the somewhat fatalistic analyses described by Hawick et al. (2017); there seems to be no way to ‘get off the carousel’, when repeated attempts at enhancement seem to reinforce a sociocultural situation that already existed.

**Figure 1:**
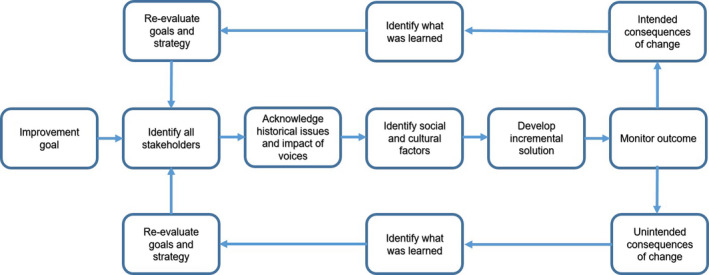
General model for wicked problem management

In the case of student attrition, the overall intended outcome of proposed enhancements is that the proportion of students progressing successfully to the end of their programme of study increases. If this overall goal is slightly reformulated, for example to say that more individuals are able to progress to the end of their programme of study, we can shift the focus to those individuals rather than thinking about the group as a whole. Whitehead (2017, p. 283) frames this kind of approach as ‘a change from putting the curriculum at the centre of attention (in a way that makes it the primary object of focus) to contextualizing the curriculum’. Using a similar approach to that proposed by DeFries and Nagendra (2017), the survey data have been used to propose a framework to inform consideration of wicked problems (Table 3; Figure 1).

**Table 3:** Examples of factors underpinning a wicked problem in healthcare student attrition and potential management questions to highlight areas of importance (developed from the approach of DeFries & Nagendra, 2017)

Factor influencing attrition	Reasons for wickedness	Useful management question
Concerns due to personal circumstances	Complex causality due to diverse student body	How do we support a changing and diverse student body? What flexibility is there within the curriculum to accommodate student needs? What mental health support is available to students?
Workload pressure	Tensions between clinical and academic demands	How can we manage multiple competing assessment deadlines? How are students helped to manage priorities?
Clinical placement culture	Variations of staff capability and motivation. Heterogeneous staff culture in different placement organisations	How do we quality assure placements? How do we ensure adequate staffing resource? How do students raise concerns?

If the overall goal is to make it possible for each student to progress, then the data presented here, along with those in many other studies of student attrition, can be used to identify unnecessary barriers to this goal and ensure that both intended and unintended consequences are considered in evaluation. This process involves setting incremental goals and reviewing them regularly; this is captured in the model shown in Figure 1.

Diverse stakeholders may have different worldviews and competing understandings (Jha & Lexa, 2014) and potential solutions to mitigate against the impacts of the wicked problem must be ongoing (Harris et al., 2009) and may be non‐linear or problematic. Actions that are successful in one context may have a different impact within another that appears on the surface to look similar and ‘waves of consequence’ may be far‐reaching and irreversible (Hannigan & Coffey, 2011, p. 221).

## 7 IMPLICATIONS

The three themes from the study presented here highlight the social and cultural complexity of the factors influencing students’ experiences. Acknowledging these as representing wickidity, and exploring student attrition using the wicked problem framework, enables us to focus on how these social factors contribute to workplace/placement culture and impact on students’ learning and personal circumstances.

Many interrelated factors determine whether an individual student withdraws from a course, and within subject areas, such as healthcare, there is variation between different areas (within and between institutions), both in terms of their retention rates and the composition of their typical learners, with large‐scale variations across institutional types and geographical location. The starting point for a wicked problem analysis, in the context of healthcare student attrition, is to consider the three systems which may influence students’ experiences: the students’ personal system, the university education system and the clinical education system. These are all in turn influenced by both local and national policy and clearly interact and influence each other.

The range of different stakeholders within the three influencing systems will all have differing perspectives, and therefore, solutions are influenced by how they frame the problem (McGrandle & Ohemeng, 2017). Collaborative strategies are needed which involve all stakeholders, and which are designed to avoid two traps: falsely assuming that a tame local solution will be effective and inaction due to the recognition of overwhelming complexity (DeFries & Nagendra, 2017).

From a university perspective, this means re‐conceptualising student attrition as a systems problem, avoiding the repetition of modest solutions and considering a more holistic approach to the problem. This reframing allows us to step back from making assumptions about blame, causality and linkages, and move from small‐scale, one‐shot, simple models to considering attrition as a complex problem derived from the interactions of factors. Without recognition of the ‘wicked’ nature of the problem, investment in interventions will continue to result in poor results and little change. It probably seems obvious that placement culture needs to be addressed in the workplace, but a multi‐system approach could also look at how students are prepared for placement and for coping with potential challenges, and how they report challenges back to university staff. It could also show how placements are integrated explicitly with other aspects of the curriculum, so that students and workplace mentors understand how they work together, and how deadlines may affect students’ activities and pressures at different times. A multi‐system approach would also anticipate challenging personal circumstances and provide flexible options for responding to these.

The question of evaluating the effectiveness of a wicked problem framework is challenging for many reasons. Notably, it can be difficult to accept the combined effect of the complex and unique nature of any particular wicked problem, and the existence of a no stopping rule, whereby even if significant improvements are made, the problem does not disappear. For example, if an institution experiences low withdrawal in a particular cohort of healthcare students, this does not mean that successful practices in that year are a guarantee of future success with student persistence on the course; the problem should be seen as dynamic and ongoing. Stakeholders need to work together to acknowledge historical issues and the impact and influence of earlier interventions, in order to move beyond previous ineffective investment in the problem of healthcare student attrition.

We have provided a framework to conceptualise healthcare student attrition based on prior research evidence. Using this as a basis for actions with diverse stakeholders should result in contextually effective approaches to the design of healthcare programmes and ultimately reduce the number of students withdrawing from these courses.
